# Oregon

**Published:** 1896-07

**Authors:** 


*Oregon.*—Oregon is second in the list with a law regulating the practice of the shoeing art, and a board consisting of three veterinary surgeons and two master horseshoers will examine all future applicants who wish to ply this vocation in this State.

